# Structure-Activity Relationship Analysis of YM155 for Inducing Selective Cell Death of Human Pluripotent Stem Cells

**DOI:** 10.3389/fchem.2019.00298

**Published:** 2019-05-16

**Authors:** Young-Hyun Go, Changjin Lim, Ho-Chang Jeong, Ok-Seon Kwon, Sungkyun Chung, Haeseung Lee, Wankyu Kim, Young-Ger Suh, Woo Sung Son, Mi-Ok Lee, Hyuk-Jin Cha, Seok-Ho Kim

**Affiliations:** ^1^Department of Life Sciences, College of Natural Sciences, Sogang University, Seoul, South Korea; ^2^Department of Pharmacy, College of Pharmacy and Institute of Pharmaceutical Sciences, CHA University, Pochen-si, South Korea; ^3^Stem Cell Convergence Research Center, Korea Research Institute of Bioscience and Biotechnology, Daejeon, South Korea; ^4^Department of Life Science, Ewha Womans University, Seoul, South Korea; ^5^College of Pharmacy, Seoul National University, Seoul, South Korea; ^6^Research Institute of Pharmaceutical Sciences, Seoul National University, Seoul, South Korea

**Keywords:** stemotoxics, naphthoquinone imidazolium, SAR (structure-activity relationship), human pluripotent stem cells, teratoma, YM155

## Abstract

Despite great potential for regenerative medicine, the high tumorigenic potential of human pluripotent stem cells (hPSCs) to form undesirable teratoma is an important technical hurdle preventing safe cell therapy. Various small molecules that induce the complete elimination of undifferentiated hPSCs, referred to as “stemotoxics,” have been developed to facilitate tumor-free cell therapy, including the Survivin inhibitor YM155. In the present work, based on the chemical structure of YM155, total 26 analogs were synthesized and tested for stemotoxic activity toward human embryonic stem cells (hESCs) and induced PSCs (iPSCs). We found that a hydrogen bond acceptor in the pyrazine ring of YM155 derivatives is critical for stemotoxic activity, which is completely lost in hESCs lacking *SLC35F2*, which encodes a solute carrier protein. These results suggest that hydrogen bonding interactions between the nitrogens of the pyrazine ring and the SLC35F2 protein are critical for entry of YM155 into hPSCs, and hence stemotoxic activity.

## Introduction

Human pluripotent stem cells (hPSCs), such as human embryonic stem cells (hESCs) and induced PSCs (iPSCs), have been actively studied due to their pluripotency, which allows them to produce all cell types in the human body and renders them a promising source for stem cell-based regenerative therapy (Trounson and Dewitt, 2016). Recent success in clinical trials of hESC-derived retinal pigment epithelial cells, for reversing loss of vision in patients suffering from age-related macular degeneration, has inspired further clinical trials on hPSCs (Schwartz et al., 2015; Song et al., 2015), including one in Japan using autologous iPSCs (Mandai et al., 2017). However, the high tumorigenic potential of undifferentiated hPSCs, due to unlimited proliferation and pluripotency leading to teratoma *in vivo*, is an important technical hurdle preventing safe stem cell therapy (Blum and Benvenisty, 2008; Ben-David and Benvenisty, 2011). Thus, a variety of techniques using small molecules (Ben-David et al., 2013; Lee et al., 2013), antibodies (Choo et al., 2008), and genetic approaches (Cho et al., 2015; Yagyu et al., 2015) have been developed to selectively eliminate or sequester undesirable residual hPSCs prior to engraftment in patients (Jeong et al., 2017a). For example, YM155 (1-(2-methoxyethyl)-2-methyl-4,9-dioxo-3-(pyrazin-2-ylmethyl)-4,9- dihydro-1H-naphtho[2,3-d]imidazol-3-ium bromide) can effectively eliminate undifferentiated hPSCs by inducing p53-dependent mitochondria cell death without affecting the functionality of differentiated cells (Lee et al., 2013). The selective cytotoxic efficacy toward undifferentiated hPSCs and safety regarding differentiated cells, termed stemotoxic activity, has been confirmed by independent studies (Bedel et al., 2017; Kang et al., 2017; Kim K. T. et al., 2017). YM155 was originally developed as an anti-cancer drug that targets Survivin (encoded by *BIRC5*) (Nakahara et al., 2007), which is highly expressed in many cancer cell types, as well as hPSCs (Lee et al., 2013). The high selectivity of YM155 toward hPSCs cannot be completely explained by suppression of *BIRC5* because p53 accumulation and consequent cell death occurs prior to dramatic suppression of *BIRC5* expression (Lee et al., 2013). Recent studies reported that YM155 is imported through solute carrier family 35 member F2 (*SLC35F2*) in cancer cells prior to induction of DNA damage, and *SLC35F2* expression determines the cytotoxicity of YM155 against cancer cells (Winter et al., 2014). Persistent DNA damage by YM155 (Wani et al., 2018b) results from redox-activated oxidative DNA damage (Wani et al., 2018a) or inhibition of topoisomerase (Hong et al., 2017), independent of the Survivin expression level (Sim et al., 2017). Analysis of the cytotoxicity of YM155 analogs in lung cancer cell lines, involving structure-activity relationship (SAR) studies on YM155, revealed that the quinone moiety and the positively charged imidazolium ring in the tricyclic naphthoimidazolium scaffold is important for cytotoxicity (Ho et al., 2015). The same analogs were also tested against two human embryonic carcinoma cell lines and compared with IMR-90 lung fibroblast cells (Ho et al., 2016). In the present study, we synthesized 26 analogs of YM155, in which the pyrazinylmethyl group was substituted with alkyl, hydroxyalkyl, aminoalkyl, substituted phenyl, and substituted benzyl groups, and we tested their stemotoxic activity toward hPSCs compared with isogenic smooth muscle cells (SMCs). We found that nitrogen in the pyrazine ring structure of YM155 serves as a hydrogen bond acceptor, and the interactions are critical for the stemotoxic activity of YM155 via uptake by SLC35F2.

## Materials and Methods

### Chemistry

#### General Information

Unless stated otherwise, all reactions were performed under argon atmosphere with dry solvents under anhydrous conditions. Tetrahydrofuran and Et_2_O were distilled immediately before use of sodium benzophenone ketyl. Dichloromethane, chloroform, triethylamine, acetonitrile, and pyridine were freshly distilled from calcium hydride. All starting materials and reagents were obtained from commercial suppliers and were used without further purification, unless otherwise noted. Solvents for routine isolation of products and chromatography were reagent grade and glass distilled. Silica gel 60 (230–400 mesh, Merck) was used for flash column chromatography. The reaction progress was monitored by thin-layer chromatography (TLC), which was performed using 0.25 mm silica gel plates (Merck). Optical rotations were measured with a JASCO P-2000 digital polarimeter at ambient temperature using 100 mm cell of 2 mL capacity. ^1^H and ^13^C NMR spectra were recorded on JEOL JNM-LA 300, BRUKER AVANCE-500, BRUKER AVANCE-400, JEOL JNM-ECA-600, and BRUKER AVANCE-800. ^1^H-NMR data were reported as follows: chemical shift (parts per million, δ), multiplicity (br, broad signal; s, singlet; d, doublet; t, triplet; q, quartet; quint, quintet; m, multiplet and/or multiple resonances), coupling constant in hertz (Hz), and number of protons. Infrared spectra were recorded on a JASCO FT-IR-4200 spectrometer and are reported in frequency of absorption (cm^−1^). High resolution mass spectra were obtained with JEOL JMS-700 instrument and Agilent Q TOF 6530.

#### Representative Synthetic Procedure of YM Analogs

##### 2-Chloro-3-((2-methoxyethyl)amino)naphthalene-1,4-dione (2)

Methoxyethylamine (2 equiv.) was added to a stirred solution of **1** and triethylamine (2 equiv.) in DCM and then stirred another 5 h. Water was added to the reaction mixture and the organic layer was separated, washed with water (2 times), and dried over MgSO_4_. Solvent was removed under reduced pressure and purified by silica gel column chromatography (ethyl acetate: hexanes = 1: 4) to afford **2** as red solid.

^1^H NMR (600 MHz, CDCl_3_) δ 8.02 (dd, *J* = 7.8, 0.9 Hz, 1H), 7.91 (d, *J* = 7.4 Hz, 1H), 7.62 (td, *J* = 7.6, 1.4 Hz, 1H), 7.53 (td, *J* = 7.6, 1.4 Hz, 1H), 6.29 (bs, 1H), 3.97 (t, *J* = 5.3 Hz, 2H), 3.56 (t, *J* = 5.4 Hz, 2H), 3.35 (s, 3H); ^13^C NMR (150 MHz, CDCl_3_) δ 180.1, 180.0, 176.5, 144.1, 134.7, 132.4, 132.3, 129.6, 126.6, 126, 5, 71.1, 71.0, 58.8, 44.3, 44.2.

##### *N*-(3-Chloro-1,4-dioxo-1,4-dihydronaphthalen-2-yl)-*N*-(2-methoxyethyl)acetamide (3)

Five drops of *c*-H_2_SO_4_ were added to a stirred solution of **2** in acetic anhydride (10 mL) and then stirred for 4 hrs. Acetic anhydride was removed under reduced pressure and purified by silica gel column chromatography (ethyl acetate: hexanes = 1: 2 to 1: 1) to afford **3** as a yellowish solid.

^1^H NMR (600 MHz, CD_3_OD) δ 8.18–8.16 (m, 2H), 7.89–7.86 (m, 2H), 7.64–7.51 (m, 2H), 4.12 (dt, *J* = 14.6, 3.7 Hz, 1H), 3.81–3.94 (m, 1H), 3.58–3.41 (m, 2H), 3.00 (s, 3H), 1.93 (s, 3H); ^13^C NMR (150 MHz, CD_3_OD) δ 181.5, 179.8, 173.1, 147.6, 143.7, 136.6, 136.5, 136.2, 133.7, 133.2, 129.0, 128.9, 72.7, 59.4, 48.7, 23.0.

##### *N*-(3-(Benzylamino)-1,4-dioxo-1,4-dihydronaphthalen-2-yl)-*N*-(2-methoxyethyl)acetamide (4c)

Triethylamine (2 equiv.) and benzylamine (2 equiv.) were added to a stirred solution of **3** in DCM and then stirred for 10 h. Water was added to the reaction mixture and the organic layer was separated, washed with water (2 times), and dried over MgSO_4_. Solvent was removed under reduced pressure and the crude material was purified by silica gel column chromatography (ethyl acetate: hexanes = 1: 1 to ethyl acetate only) to afford **4c** as a red oil.

^1^H NMR (500 MHz, DMSO-d_6_) δ 7.98 (d, *J* = 8.0 Hz, 1H), 7.93–7.89 (m, 2H), 7.79 (td, *J* = 7.7, 1.2 Hz, 1H), 7.71 (td, *J* = 7.5, 1.2 Hz, 1H), 7.27 (t, *J* = 7.5 Hz, 1H), 7.20–7.18 (m, 3H), 4.67–4.56 (m, 2H), 3.76 (bs, 1H), 3.42–3.38 (m, 1H), 3.31 (s, 1H), 3.21–3.08 (m, 2H), 3.00 (s, 3H); ^13^C NMR (125 MHz, DMSO-d_6_) δ 182.8, 170.0, 172.0, 144.0, 140.0, 135.5, 133.2, 132.5, 131.0, 128.9, 127.5, 127.1, 127.0, 126.3, 117.7, 69.4, 58.3, 47.6, 47.0, 21.5.

##### 3-Benzyl-1-(2-methoxyethyl)-2-methyl-4,9-dioxo-4,9-dihydro-1*H*-naphtho[2,3-*d*]imidazol-3-ium bromide (5c)

c-HBr (2 equiv.) was added to a stirred solution of **4c** in EtOH and then refluxed for 12 h. EtOH was removed under reduced pressure and ethyl acetate was added and stirred overnight. Precipitate formed was filtered and additional purification was performed by silica gel column chromatography (DCM: MeOH = 10: 1) to afford **5c** as a yellow solid.

##### 1-(2-Methoxyethyl)-2-methyl-4,9-dioxo-3-(4-methoxyphenyl)-4,9-dihydro-1*H*-naphtho[2,3-*d*]imidazol-3-ium bromide (5a)

^1^H NMR (300 MHz, CDCl_3_) δ 8.27 (m, 1H), 8.03 (m, 1H), 7.84–7.72 (m, 4H), 7.10–7.04 (m, 2H), 4.94 (t, *J* = 4.8 Hz, 2H), 3.90 (t, *J* = 4.8 Hz, 2H), 3.88 (s, 3H), 3.33 (s, 3H), 2.85 (s, 3H); ^13^C NMR (125 MHz, CD_3_OD) δ 174.9, 174.5, 157.4, 154.2, 134.9, 131.9, 130.7, 130.4, 129.4, 126.8, 120.8, 120.5, 110.8, 69.9, 58.0, 54.7, 47.9, 47.1, 10.2.

##### 3-(3,4-dimethoxyphenyl)-1-(2-methoxyethyl)-2-methyl-4,9-dioxo-4,9-dihydro-1H-naphtho[2,3-d]imidazol-3-ium bromide (5b)

^1^H NMR (500 MHz, CD_3_OD) δ 8.26–8.10 (m, 2H), 7.89 (bs, 2H), 7.32 (bs, 1H), 7.18 (bs, 2H), 4.94 (bs, 2H), 3.93–3.84 (m, 8H), 3.88 (s, 3H), 3.36 (s, 3H), 2.68 (s, 3H); ^13^C NMR (125 MHz, CD_3_OD) δ 175.1, 173.1, 154.3, 151.5, 150.0, 134.9, 134.7, 132.0, 131.5, 126.8, 126.7, 125.3, 119.3, 111.4, 110.3, 69.9, 58.1, 55.6, 55.4, 48.3, 10.3.

##### 3-Benzyl-1-(2-methoxyethyl)-2-methyl-4,9-dioxo-4,9-dihydro-1*H*-naphtho[2,3-*d*]imidazol-3-ium bromide (5c)

^1^H NMR (600 MHz, CD_3_OD) δ 8.27–8.24 (m, 2H), 7.96–7.92 (m, 2H), 7.42–7.34 (m, 5H), 6.06 (s, 2H), 4.93 (t, *J* = 4.8 Hz, 2H), 3.86 (t, *J* = 4.8 Hz, 2H), 3.30 (s, 3H), 2.82 (m, 3H); ^13^C NMR (150 MHz, CD_3_OD) δ 176.3, 176.2, 155.2, 136.3, 136.2, 134.5, 133.4, 133.4, 131.9, 131.7, 130.4, 129.9, 128.3, 128.3, 128.2, 71.1, 59.4, 51.5, 49.4, 10.8; HR-MS (ESI+) calcd for C_22_H_21_N_2_O_3_ [M – Br^−^]^+^ 361.1547, found 361.1527.

##### 3-(2-Fluorobenzyl)-1-(2-methoxyethyl)-2-methyl-4,9-dioxo-4,9-dihydro-1*H*-naphtho[2,3-*d*]imidazol-3-ium bromide (5d)

^1^H NMR (600 MHz, CDCl_3_) δ 8.27–8.25 (m, 1H), 8.23–8.22 (m, 1H), 7.96–7.91 (m, 2H), 7.43 (td, *J* = 7.8, 7.3 Hz, 1H), 7.36 (t, *J* = 7.8 Hz, 1H), 7.23–7.19 (m, 2H), 6.10 (s, 2H), 4.95 (t, *J* = 5.0 Hz, 2H), 3.87 (t, *J* = 5.0 Hz, 2H), 3.32 (s, 3H), 2.90 (s, 3H); ^13^C NMR (150 MHz, CD_3_OD) δ 176.2, 176.0, 161.9 (d, *J*_C−F_ = 244.9 Hz), 155.5, 136.3, 136.3, 133.3 (d, *J*_C−F_ = 15.8 Hz), 132.3, 132.3, 132.0, 131.7, 130.6 (d, *J*_C−F_ = 2.9 Hz), 128.3, 128.3, 126.1 (d, *J*_C−F_ = 3.6 Hz), 121.5 (d, *J*_C−F_ = 13.7 Hz), 117.0 (d, *J*_C−F_ = 20.8 Hz), 71.1, 59.4, 49.6, 46.6, 11.3; HR-MS (ESI+) calcd for C_22_H_20_FN_2_O_3_ [M – Br^−^]^+^ 379.1452, found 379.1440.

##### 3-(3-Fluorobenzyl)-1-(2-methoxyethyl)-2-methyl-4,9-dioxo-4,9-dihydro-1*H*-naphtho[2,3-*d*]imidazol-3-ium bromide (5e)

^1^H NMR (300 MHz, CD_3_OD) δ 8.38–8.23 (m, 2H), 8.02–7.86 (m, 2H), 7.54–7.32 (m, 1H), 7.32–6.98 (m, 3H), 6.07 (s, 2H), 4.94 (t, *J* = 4.9 Hz, 2H), 3.87 (t, *J* = 4.9 Hz, 2H), 3.32 (s, 3H), 2.85 (m, 3H); ^13^C NMR (75 MHz, CD_3_OD) δ 176.3, 176.1, 164.5 (d, *J*_C−F_ = 246.3 Hz), 155.2, 137.0 (d, *J*_C−F_ = 7.6 Hz), 136.3, 136.2, 133.3, 133.3, 132.3 (d, *J*_C−F_ = 8.4 Hz), 131.9, 131.6, 128.2, 124.1 (d, *J*_C−F_ = 3.0 Hz), 116.7 (d, *J*_C−F_ = 21.2 Hz), 115.2 (d, *J*_C−F_ = 23.2 Hz), 71.1, 59.4, 50.9, 49.4, 11.4; HR-MS (ESI+) calcd for C_22_H_20_FN_2_O_3_ [M – Br^−^]^+^ 379.1452, found 379.1470.

##### 3-(4-Fluorobenzyl)-1-(2-methoxyethyl)-2-methyl-4,9-dioxo-4,9-dihydro-1*H*-naphtho[2,3-*d*]imidazol-3-ium bromide (5f)

^1^H NMR (300 MHz, DMSO-d_6_) δ 8.22–8.16 (m, 2H), 8.04–7.99 (m, 2H), 7.42 (dd, *J* = 8.9, 5.6 Hz, 2H), 7.25 (t, *J* = 9.0 Hz, 2H), 6.02 (s, 2H), 4.86 (t, *J* = 4.8 Hz, 2H), 3.79 (t, *J* = 4.8 Hz, 2H), 3.23 (s, 3H), 2.86 (s, 3H); ^13^C NMR (75 MHz, DMSO-d_6_) δ 174.9, 174.8, 162.0 (d, *J*_C−F_ = 243.3 Hz), 153.3, 135.3, 135.3, 131.7, 131.7, 130.5, 130.0, 129.8 (d, *J*_C−F_ = 3.2 Hz), 129.4 (d, *J*_C−F_ = 8.3 Hz), 126.9, 115.8 (d, *J*_C−F_ = 21.7 Hz), 69.5, 58.5, 48.9, 47.5, 10.7; HR-MS (ESI+) calcd for C_22_H_20_FN_2_O_3_ [M – Br^−^]^+^ 379.1452, found 379.1469.

##### 3-(4-Chlorobenzyl)-1-(2-methoxyethyl)-2-methyl-4,9-dioxo-4,9-dihydro-1H-naphtho[2,3-d]imidazol-3-ium bromide (5g)

^1^H NMR (300 MHz, CD_3_OD) δ 8.30–8.21 (m, 2H), 7.98–7.90 (m, 2H), 7.43–7.36 (m, 4H), 6.04 (s, 2H), 4.93 (t, *J* = 4.9 Hz, 2H), 3.86 (t, *J* = 4.8 Hz, 2H), 3.31 (s, 3H), 2.86 (m, 3H); ^13^C NMR (75 MHz, CD_3_OD) δ 176.3, 176.2, 155.2, 136.3, 136.3, 135.8, 133.4, 133.3, 133.2, 132.0, 131.6, 130.4, 130.1, 128.3, 71.1, 59.4, 50.9, 49.4, 11.1; HR-MS (ESI+) calcd for C_22_H_20_ClN_2_O_3_ [M – Br^−^]^+^ 395.1157, found 395.1182.

##### 3-(2-Methoxybenzyl)-1-(2-methoxyethyl)-2-methyl-4,9-dioxo-4,9-dihydro-1*H*-naphtho[2,3-*d*]imidazol-3-ium bromide (5h)

^1^H NMR (400 MHz, CD_3_OD) δ 8.27–8.21 (m, 2H), 7.95–7.91 (m, 2H), 7.37 (td, *J* = 7.9, 1.5 Hz, 1H), 7.30 (d, *J* = 7.3 Hz, 1H), 7.04 (d, *J* = 8.2 Hz, 1H), 6.97 (t, *J* = 7.5 Hz, 1H), 5.98 (s, 2H), 4.92 (t, *J* = 4.8 Hz, 2H), 3.86 (t, *J* = 4.8 Hz, 2H), 3.81 (s, 3H), 3.31 (s, 3H), 2.84 (m, 3H); ^13^C NMR (150 MHz, CD_3_OD) δ 176.2, 175.9, 158.8, 155.5, 136.2, 133.3, 132.1, 131.8, 131.8, 130.8, 128.2, 128.2, 122.1, 121.9, 112.2, 71.3, 59.4, 56.1, 49.5, 48.4, 10.9; HR-MS (ESI+) calcd for C_23_H_23_N_2_O_4_ [M – Br^−^]^+^ 391.1652, found 391.1631.

##### 3-(3-Methoxybenzyl)-1-(2-methoxyethyl)-2-methyl-4,9-dioxo-4,9-dihydro-1H-naphtho[2,3-d]imidazol-3-ium bromide (5i)

^1^H NMR (400 MHz, CD_3_OD) δ 8.26 (td, *J* = 7.5, 4.1 Hz, 2H), 8.01–7.87 (m, 2H), 7.31 (td, *J* = 7.6, 1.1 Hz, 1H), 6.95–6.89 (m, 1H), 6.91 (s, 1H), 6.86 (d, *J* = 7.7 Hz, 1H), 6.03 (s, 2H), 4.93 (t, *J* = 4.8 Hz, 2H), 3.86 (t, *J* = 4.9 Hz, 2H), 3.78 (s, 3H), 3.31 (s, 3H), 2.83 (s, 3H); ^13^C NMR (100 MHz, CD_3_OD) δ 176.3, 176.2, 161.8, 155.2, 136.3, 136.2, 135.9, 133.4, 131.9, 131.6, 131.5, 128.3, 128.2, 120.1, 115.1, 114.2, 71.1, 59.4, 55.9, 51.3, 49.4, 10.9; HR-MS (ESI+) calcd for C_23_H_23_N_2_O_4_ [M – Br^−^]^+^ 391.1652, found 391.1629.

##### 3-(4-Methoxybenzyl)-1-(2-methoxyethyl)-2-methyl-4,9-dioxo-4,9-dihydro-1H-naphtho[2,3-d]imidazol-3-ium bromide (5j)

^1^H NMR (800 MHz, CDCl_3_) δ 8.19–8.14 (m, 2H), 7.82 (dd, *J* = 5.7, 3.3 Hz, 2H), 7.25 (d, *J* = 8.8 Hz, 2H), 6.85 (d, *J* = 8.8 Hz, 2H), 6.00 (s, 2H), 4.93 (t, *J* = 4.9 Hz, 2H), 3.78 (t, *J* = 5.0 Hz, 2H), 3.73 (s, 3H), 3.23 (s, 3H), 3.03 (s, 3H); ^13^C NMR (200 MHz, CDCl_3_) δ 174.7, 174.7, 160.0, 155.0, 152.0, 135.2, 135.2, 131.6, 130.2, 130.1, 129.0, 127.6, 127.4, 124.4, 114.6, 69.8, 59.1, 55.3, 50.9, 48.8, 13.2; HR-MS (ESI+) calcd for C_23_H_23_N_2_O_4_ [M – Br^−^]^+^ 391.1652, found 391.1631.

##### 1-(2-Methoxyethyl)-2-methyl-4,9-dioxo-3-(pyridin-2-ylmethyl)-4,9-dihydro-1*H*-naphtho[2,3-*d*]imidazol-3-ium bromide (5k)

^1^H NMR (800 MHz, DMSO-d_6_) δ 8.53 (d, *J* = 4.6 Hz, 1H), 8.17 (dd, *J* = 7.6, 0.9 Hz, 1H), 8.09 (dd, *J* = 7.5, 1.0 Hz, 1H), 8.01–7.94 (m, 3H), 7.70 (d, *J* = 8.0 Hz, 1H), 7.46 (dd, *J* = 7.1, 5.3 Hz, 1H), 6.15 (s, 2H), 4.92 (t, *J* = 4.9 Hz, 2H), 3.82 (t, *J* = 4.9 Hz, 2H), 3.26 (s, 3H), 2.97 (s, 3H); ^13^C NMR (200 MHz, CD_3_OD) δ 174.7, 174.6, 154.2, 152.0, 148.6, 138.7, 135.4, 135.2, 131.8, 131.4, 130.3, 130.0, 127.0, 126.8, 124.0, 122.8, 69.6, 58.6, 48.6, 47.7, 11.0; HR-MS (ESI+) calcd for C_21_H_20_N_3_O_3_ [M – Br^−^]^+^ 362.1499, found 362.1476.

##### 1-(2-Methoxyethyl)-2-methyl-4,9-dioxo-3-(pyridin-3-ylmethyl)-4,9-dihydro-1*H*-naphtho[2,3-*d*]imidazol-3-ium bromide (5l)

^1^H NMR (600 MHz, CD_3_OD) δ 8.65 (d, *J* = 2.2 Hz, 1H), 8.56 (dd, *J* = 4.9, 1.4 Hz, 1H), 8.29–8.23 (m, 2H), 7.97–7.91 (m, 2H), 7.87 (dd, *J* = 6.5, 1.7 Hz, 1H), 7.48 (dd, *J* = 8.1, 4.9 Hz, 1H), 6.09 (s, 2H), 4.94 (t, *J* = 4.9 Hz, 2H), 3.87 (t, *J* = 4.9 Hz, 2H), 3.32 (s, 3H), 2.91 (s, 3H); ^13^C NMR (150 MHz, CD_3_OD) δ 176.3, 176.2, 155.3, 150.6, 149.4, 137.4, 136.4, 136.3, 133.4, 133.3, 132.0, 131.7, 131.3, 128.3, 128.3, 125.7, 71.1, 59.4, 49.5, 49.3, 10.8; HR-MS (ESI+) calcd for C_21_H_20_N_3_O_3_ [M – Br^−^]^+^ 362.1499, found 362.1488.

##### 1-(2-Methoxyethyl)-2-methyl-4,9-dioxo-3-(pyridin-4-ylmethyl)-4,9-dihydro-1*H*-naphtho[2,3-*d*]imidazol-3-ium bromide (5m)

^1^H NMR (800 MHz, DMSO-d_6_) δ 8.76 (br s, 2H), 8.22 (dd, *J* = 7.6, 1.2 Hz, 1H), 8.11 (dd, *J* = 7.5, 1.3 Hz, 1H), 8.02 (td, *J* = 7.5, 1.4 Hz, 1H), 7.99 (td, *J* = 7.5, 1.4 Hz, 1H), 7.58 (s, 2H), 6.15 (s, 2H), 4.89 (t, *J* = 5.0 Hz, 2H), 3.81 (t, *J* = 5.0 Hz, 2H), 3.26 (s, 3H), 2.86 (s, 3H); ^13^C NMR (200 MHz, DMSO-d_6_) δ 174.8, 174.7, 153.9, 147.0, 135.4, 135.3, 131.8, 131.5, 130.5, 130.1, 127.0, 126.8, 122.7, 122.6, 69.5, 58.5, 49.0, 47.7, 10.5; HR-MS (ESI+) calcd for C_21_H_20_N_3_O_3_ [M – Br^−^]^+^ 362.1499, found 362.1532.

##### 3-(4-Hydroxyphenethyl)-1-(2-methoxyethyl)-2-methyl-4,9-dioxo-4,9-dihydro-1H-naphtho[2,3-d]imidazol-3-ium bromide (5n)

^1^H NMR (800 MHz, DMSO-d_6_) δ 9.36 (s, 1H), 8.21 (br s, 2H), 8.02 (br s, 2H), 7.01 (d, *J* = 8.4 Hz, 2H), 6.69 (d, *J* = 8.4 Hz, 2H), 4.79 (br s, 2H), 3.72 (t, *J* = 4.9 Hz, 2H), 3.34 (s, 3H), 3.24 (s, 3H), 3.02 (t, *J* = 6.7 Hz, 2H), 2.41 (br s, 2H); ^13^C NMR (200 MHz, DMSO-d_6_) δ 174.8, 174.6, 156.5, 152.5, 135.2, 135.2, 131.8, 131.6, 130.3, 130.0, 126.9, 126.5, 115.4, 69.6, 58.5, 49.2, 47.3, 33.7, 9.8; HR-MS (ESI+) calcd for C_23_H_23_N_2_O_4_ [M – Br^−^]^+^ 391.1652, found 391.1637.

##### 1-(2-Methoxyethyl)-3-(4-methoxyphenethyl)-2-methyl-4,9-dioxo-4,9-dihydro-1H-naphtho[2,3-d]imidazol-3-ium bromide (5o)

^1^H NMR (800 MHz, CD_3_OD) δ 8.30-8.23 (m, 2H), 8.01–7.86 (m, 2H), 7.12 (dd, *J* = 8.4, 3.5 Hz, 2H), 6.84 (d, *J* = 8.5 Hz, 2H), 4.88 (t, *J* = 6.8 Hz, 2H), 4.84 (t, *J* = 5.3 Hz, 2H), 3.80 (t, *J* = 4.7 Hz, 2H), 3.73 (s, 3H), 3.34 (s, 3H), 3.31 (s, 3H), 3.16 (t, *J* = 6.8 Hz, 2H); ^13^C NMR (200 MHz, CD_3_OD) δ 176.2, 176.0, 160.6, 154.5, 136.2, 136.2, 133.4, 133.3, 131.7, 131.7, 131.5, 129.5, 128.3, 128.2, 115.5, 71.2, 59.4, 55.7, 50.8, 49.8, 35.5, 10.2; HR-MS (ESI+) calcd for C_24_H_25_N_2_O_4_ [M – Br^−^]^+^ 405.1809, found 405.1825.

##### 3-(Furan-2-ylmethyl)-1-(2-methoxyethyl)-2-methyl-4,9-dioxo-4,9-dihydro-1*H*-naphtho[2,3-*d*]imidazol-3-ium bromide (5p)

^1^H NMR (500 MHz, DMSO-d_6_) δ 8.22 (br s, 1H), 8.18 (br s, 1H), 8.01 (br s, 2H), 7.72 (d, *J* = 1.0 Hz, 1H), 6.71 (d, *J* = 3.2 Hz, 1H), 6.50 (dd, *J* = 3.1, 1.9 Hz, 1H), 6.04 (s, 2H), 4.85 (br s, 2H), 3.79 (t, *J* = 4.9 Hz, 2H), 3.24 (s, 3H), 3.01 (s, 3H); ^13^C NMR (125 MHz, DMSO-d_6_) δ 174.6, 174.5, 153.1, 146.4, 144.3, 135.3, 135.2, 131.7, 131.4, 130.4, 129.4, 126.9, 126.8, 111.0, 110.9, 69.4, 58.4, 47.5, 43.1, 10.5; HR-MS (ESI+) calcd for C_20_H_19_N_2_O_4_ [M – Br^−^]^+^ 351.1339, found 351.1326.

##### 1-(2-Methoxyethyl)-2-methyl-4,9-dioxo-3-(thiophen-2-ylmethyl)-4,9-dihydro-1*H*-naphtho[2,3-*d*]imidazol-3-ium bromide (5q)

^1^H NMR (400 MHz, CD_3_OD) δ 8.32-8.30 (m, 1H), 8.27–8.25 (m, 1H), 7.97–7.94 (m, 2H), 7.48 (dd, *J* = 5.2, 1.1 Hz, 1H), 7.37 (d, *J* = 3.5 Hz, 1H), 7.04 (dd, *J* = 5.1, 3.6 Hz, 1H), 6.22 (s, 2H), 4.90 (t, *J* = 4.9 Hz, 2H), 3.84 (t, *J* = 4.9 Hz, 2H), 3.30 (s, 3H), 3.02 (s, 3H); ^13^C NMR (100 MHz, CD_3_OD) δ 176.4, 176.1, 154.8, 136.4, 136.3, 136.0, 133.4, 133.3, 131.9, 131.0, 130.5, 128.9, 128.3, 128.3, 128.3, 71.1, 59.3, 49.4, 46.6, 11.5; HR-MS (ESI+) calcd for C_20_H_19_N_2_O_3_S [M – Br^−^]^+^ 367.1111, found 367.1091.

##### 3-(2-Hydroxyethyl)-1-(2-methoxyethyl)-2-methyl-4,9-dioxo-4,9-dihydro-1*H*-naphtho[2,3-*d*]imidazol-3-ium bromide (5r)

^1^H NMR (800 MHz, CD_3_OD) δ 8.27–8.25 (m, 2H), 7.95–7.93 (m, 2H), 4.92 (t, *J* = 4.9 Hz, 2H), 4.83 (t, *J* = 5.1 Hz, 2H), 4.00 (t, *J* = 5.0 Hz, 2H), 3.86 (t, *J* = 5.0 Hz, 2H), 3.32 (s, 3H), 2.92 (s, 3H); ^13^C NMR (200 MHz, CD_3_OD) δ 176.3, 176.2, 155.6, 136.2, 133.3, 133.3, 131.8, 131.8, 128.2, 128.2, 71.3, 60.9, 59.4, 51.4, 49.2, 11.2; HR-MS (ESI+) calcd for C_17_H_19_N_2_O_4_ [M – Br^−^]^+^ 315.1339, found 315.1326.

##### 3-(3-Hydroxypropyl)-1-(2-methoxyethyl)-2-methyl-4,9-dioxo-4,9-dihydro-1*H*-naphtho[2,3-*d*]imidazol-3-ium bromide (5s)

^1^H NMR (400 MHz, CD_3_OD) δ 8.27–8.22 (m, 2H), 7.96-7.92 (m, 2H), 4.91 (t, *J* = 4.9 Hz, 2H), 4.82 (t, *J* = 7.0 Hz, 2H), 3.86 (t, *J* = 4.9 Hz, 2H), 3.70 (t, *J* = 5.7 Hz, 2H), 3.33 (s, 3H), 2. 97 (s, 3H), 2.15 (dq, *J* = 6.6, 6.1 Hz, 2H); ^13^C NMR (100 MHz, CD_3_OD) δ 176.3, 176.1, 154.7, 136.2, 136.1, 133.3, 133.3, 131.9, 131.7, 128.2, 128.1, 71.2, 59.5, 59.4, 48.8, 46.9, 34.7, 11.2; HR-MS (ESI+) calcd for C_18_H_21_N_2_O_4_ [M – Br^−^]^+^ 329.1496, found 329.1493.

##### 3-(4-Hydroxybutyl)-1-(2-methoxyethyl)-2-methyl-4,9-dioxo-4,9-dihydro-1*H*-naphtho[2,3-*d*]imidazol-3-ium bromide (5t)

^1^H NMR (300 MHz, CD_3_OD) δ 8.30–8.23 (m, 2H), 7.97–7.93 (m, 2H), 4.91 (t, *J* = 4.8 Hz, 2H), 4.75 (t, *J* = 7.8 Hz, 2H), 3.83 (t, *J* = 4.9 Hz, 2H), 3.65 (t, *J* = 6.2 Hz, 2H), 3.33 (s, 3H), 2.95 (s, 3H), 2.04–1.94 (m, 2H), 1.74–1.65 (m, 2H); ^13^C NMR (75 MHz, CD_3_OD) δ 176.3, 176.1, 154.3, 136.2, 136.2, 133.3, 131.8, 128.2, 128.2, 71.2, 62.1, 59.4, 49.2, 30.1, 27.3, 11.2; HR-MS (ESI+) calcd for C_19_H_23_N_2_O_4_ [M – Br^−^]^+^ 343.1652, found 343.1658.

##### 3-(5-Hydroxypentyl)-1-(2-methoxyethyl)-2-methyl-4,9-dioxo-4,9-dihydro-1*H*-naphtho[2,3-*d*]imidazol-3-ium bromide (5u)

^1^H NMR (800 MHz, DMSO-d_6_) δ 8.20–8.18 (m, 2H), 8.01–8.00 (m, 2H), 4.84 (t, *J* = 4.9 Hz, 2H), 4.65 (t, *J* = 7.6 Hz, 2H), 4.44 (t, *J* = 5.2 Hz, 1H), 3.78 (t, *J* = 5.0 Hz, 2H), 3.40 (td, *J* = 6.2, 5.4 Hz, 2H), 3.25 (s, 3H), 2.93 (s, 3H), 1.81 (quint, *J* = 7.7 Hz, 2H), 1.50–1.46 (m, 2H), 1.42–1.39 (m, 2H); ^13^C NMR (200 MHz, DMSO-d_6_) δ 174.8, 174.6, 152.7, 135.2, 135.2, 131.6, 131.6, 130.2, 130.0, 126.9, 126.8, 69.6, 60.4, 58.5, 47.3, 31.9, 28.7, 22.3, 10.4; LR-MS (FAB+) *m/z* 357 [M – Br^−^]^+^; HR-MS (FAB+) calcd for C_20_H_25_N_2_O_4_ [M – Br^−^]^+^ 357.1814, found 357.1818.

##### 3-(6-hydroxyhexyl)-1-(2-methoxyethyl)-2-methyl-4,9-dioxo-4,9-dihydro-1H-naphtho[2,3-d]imidazol-3-ium bromide (5v)

^1^H NMR (500 MHz, CD_3_OD) δ 8.25–8.22 (m, 2H), 7.93-7.91 (m, 2H), 4.88 (t, *J* = 4.9 Hz, 2H), 4.68 (t, *J* = 8.0 Hz, 2H), 3.84 (t, *J* = 6.6 Hz, 2H), 3.30 (s, 3H), 2.90 (s, 3H), 1.90 (quint, *J* = 7.2 Hz, 2H), 1.56 (quint, *J* = 6.9 Hz, 2H), 1.51–1.45 (m, 4H); ^13^C NMR (125 MHz, CD_3_OD) δ 174.9, 174.7, 153.0, 134.8, 134.8, 132.0, 132.0, 130.4, 126.8, 126.8, 69.9, 61.3, 58.0, 32.0, 29.1, 25.8, 25.0, 9.6.

##### 3-(2-Aminoethyl)-1-(2-methoxyethyl)-2-methyl-4,9-dioxo-4,9-dihydro-1H-naphtho[2,3-d]imidazol-3-ium bromide (5w)

^1^H NMR (500 MHz, DMSO-d_6_) δ 8.18–8.16 (m, 2H), 8.10 (bs, 3H), 8.00–7.98 (m, 2H), 4.84 (t, *J* = 4.9 Hz, 2H), 4.83 (t, *J* = 4.1 Hz, 2H), 3.74 (t, *J* = 4.7 Hz, 2H), 3.36 (m, 2H), 3.22 (s, 3H), 2.91 (s, 3H).

##### 1-(3-Aminopropyl)-3-(2-methoxyethyl)-4,9-dioxo-4,9-dihydro-1H-naphtho[2,3-d]imidazol-3-ium bromide (5x)

^1^H NMR (500 MHz, DMSO-d_6_) δ 8.17–8.13 (m, 2H), 8.00–7.95 (m, 2H), 7.87 (bs, 2H), 4.81–78 (m, 2H), 4.68 (t, *J* = 7.7 Hz, 1.4H), 4.62 (t, *J* = 7.5 Hz, 1.4H), 3.75–3.71 (m, 2H), 3.22 (s, 3H), 3.12 (q, *J* = 6.1 Hz, 0.6H), 2.97–2.93 (m, 1.4H), 2.88 (s, 3H), 2.08 (quint, *J* = 7.6 Hz, 1.4H), 1.91 (t, *J* = 7.4 Hz, 0.6H).

##### 1,3-Bis(2-methoxyethyl)-4,9-dioxo-4,9-dihydro-1H-naphtho[2,3-d]imidazol-3-ium bromide (5y)

^1^H NMR (300 MHz, CD_3_OD) δ 8.27 (dd, *J* = 5.8, 3.4 Hz, 2H), 7.94 (dd, *J* = 5.9, 3.3 Hz, 2H), 4.92 (t, *J* = 4.8 Hz, 4H), 3.85 (t, *J* = 4.8 Hz, 4H), 3.31 (s, 6H), 2.91 (s, 3H); ^13^C NMR (75 MHz, CD_3_OD) δ 176.3, 155.7, 136.2, 133.3, 131.7, 128.2, 71.3, 59.4, 49.2, 11.5; HR-MS (ESI+) calcd for C_19_H_23_N_2_O_4_ [M – Br^−^]^+^ 392.1496, found 392.1504.

##### 1-Butyl-3-(2-methoxyethyl)-4,9-dioxo-4,9-dihydro-1H-naphtho[2,3-d]imidazol-3-ium bromide (5z)

^1^H NMR (800 MHz, CD_3_OD) δ 8.27–8.25 (m, 2H), 7.95–7.94 (m, 2H), 4.90 (t, *J* = 4.9 Hz, 2H), 4.71 (t, *J* = 7.9 Hz, 2H), 3.86 (t, *J* = 4.9 Hz, 2H), 3.33 (s, 3H), 2.92 (s, 3H), 1.92–1.86 (m, 2H), 1.53 (sext, *J* = 7.5 Hz, 2H), 1.04 (t, *J* = 7.4 Hz, 3H); ^13^C NMR (200 MHz, CD_3_OD) δ 176.3, 176.1, 154.3, 136.2, 136.2, 133.4, 133.3, 131.7, 131.7, 128.2, 128.2, 71.2, 59.4, 49.2, 48.9, 32.4, 20.7, 13.9, 11.0; HR-MS (ESI+) calcd for C_19_H_23_N_2_O_3_ [M – Br^−^]^+^ 327.1706, found 327.1703.

### hPSCs and Differentiated Cell Culture

Human ESCs (H9, Wicell Research Institute) and hiPSCs (SES8) (Lee et al., 2010) were maintained in ESC medium (Stem Cell Technology, # 29106). For treatment of YM155 and YM155 analogs, hESCs or hiPSCs were cultured in mTeSR1 medium (with 50× supplement, 0.1% gentamycin) on Matrigel (Corning, # 354277)-coated 60mm tissue culture dishes (Corning, # 430166) or tissue culture 6, 12, 24-well plate (Falcon, # 353046, # 353043, # 353047), as described previously (Cho et al., 2015, 2016, 2018; Jeong et al., 2017b; Kim S. Y. et al., 2017). Smooth muscle cells (hASMC and SMC3) (Lee et al., 2010) were cultured in SMCM medium (ScienCell Research Laboratories, # 1101).

### Cell Death Assay

For Annexin V staining, cells with YM155 or YM155 analogs treated for 24 h, were washed twice with PBS and were stained with FITC-Annexin V (BD Bioscience, # 556419) and 7-AAD (BD Bioscience, # 559925) or PE-Annexin V (BD Bioscience, # 556421) and 7-AAD for 30 min at RT in the dark. Cell death was analyzed with FACS as described previously (BD Bioscience) (Kwon et al., 2017). Cells stained with Annexin / 7-AAD were analyzed by FACS Calibur I (BD Bioscience). For all of the cell images captured, Light channel of optical microscope (Olympus, CKX-41) or JULI-stage (NanoEntek) were used according to the manufacture's protocol.

### Immunoblotting Assay

Immunoblotting assay were performed as described previously (Kwon et al., 2017). Antibodies used in this study, PARP-1 (# sc-7150) and α-tubulin (# sc-8035), were purchased from Santa Cruz Biotechnology. Antibodies for cleaved caspase-3 (#9664S) were purchased from Cell Signaling Biotechnology.

### Structure Optimization and Electrostatic Surface Potential Analysis

MarvinSketch was used for drawing, displaying and characterizing chemical structures, substructures, and reactions, MarvinSketch 17.28, 2018, ChemAxon (http://www.chemaxon.com). The YM155 and its analogs were prepared in the form of 3D structures with Chem3D software (http://www.perkinelmer.com) that immediately converted the 2D chemical structures into 3D structures. Initially, energy minimization of 3D structures was done with MM2 force field in Chem3D. For further refinement, minimization was performed with UFF/GAFF force field in Avogadro (Hanwell et al., 2012). The Coulombic surface coloring method with UCSF Chimera was used to calculate the electrostatic surface potentials of the YM155 analogs. All structures were represented with UCSF Chimera (Pettersen et al., 2004; Goddard et al., 2005).

### Statistical Analysis

The graphical data were shown as mean ± SEM. Statistical significance between two or three groups were determined by using ordinary one-way or two-way ANOVA after Bonferroni posttest, respectively. Significance was assumed for *P* < 0.05 (^*^), 0.01 (^**^), 0.001 (^***^) and 0.0001 (^****^).

## Results and Discussions

### Synthesis of YM Analogs and Requirement of the Imidazolium Ring Structure for Stemotoxic Activity

**Figure 1:**
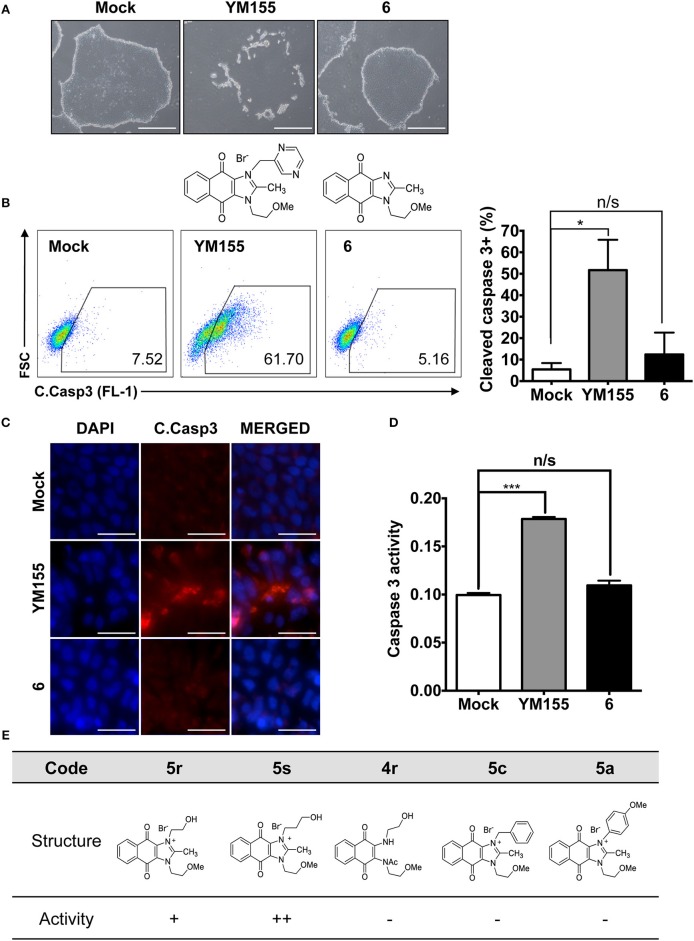
Requirement of the imidazolium ring structure for stemotoxic activity **(A)** Light microscopic images of hiPSCs after treatment of YM155 (50 nM) and **6** (50 nM) for 24 h (top), Structure of YM155 and **6** (bottom) (Scale bar = 300 μm). **(B)** FACS analysis for the cleaved caspase3 (C.Casp3) staining (left), Percentage of cleaved caspase 3 positive cells were presented as bar graph (right) (*n* = 3) (*P* < 0.05 (*)). **(C)** Fluorescence microscopic images of hiPSCs 24 h after 50 nM of YM155 or **6**, stained for cleaved caspase3 (C.Casp3) with DAPI nuclear counterstaining (Scale bar = 50 μm). **(D)** Relative enzyme activity of caspase3 after treatment of indicated chemicals (*n* = 3) (*P* < 0.05 (***)) **(E)** Summary table of three YM155 analogs with chemical structures.

**Scheme 1:**
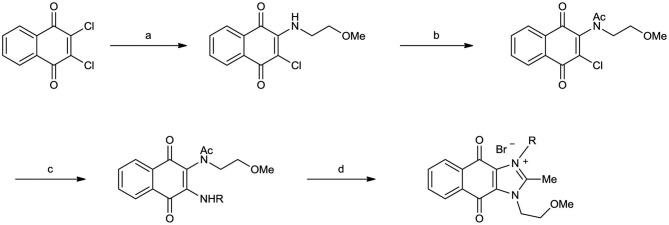
Synthetic procedure of YM155 analogs *Reagents and conditions* **(a)** Et_3_N, CH_2_Cl_2_, r.t. **(b)** Ac_2_O, c-H_2_SO_4_, 80°C **(c)** RNH_2_, Et_3_N, CH_2_Cl_2_, r.t. or CHCl_3_, reflux (for **5a**, **5b**) **(d)** HBr, EtOH, reflux.

Naphthoquinone imidazolium YM155 analogs were synthesized based on the procedure shown in Scheme 1. Dichloronaphthoquinone (**1**) was reacted with 2-methoxyethylamine in the presence of triethylamine to afford **2**, and subsequently acetylated to afford **3**. Reaction of **3** with diverse amines afforded **4**, and compounds were cyclized in the presence of HBr in refluxing EtOH to afford naphthoquinone imidazolium salt **5**. Compound **6** was synthesized according to the procedure reported previously (Kuo et al., 1996). Structures of synthesized compounds are listed in Table 1. Through initial screening of 30 YM155 analogs based on cellular morphology, YM155 analogs with stemotoxic activity were selected (Figure S1). In particular, loss of the pyrazine moiety of YM155 (shown as **6**) results in the clear reduction of stemotoxic activity toward hESCs based on cellular morphology (Figure 1A). The significance of pyrazine moiety of YM155 in stemotoxic activity was confirmed by the level of active caspase 3 formation (Figures 1B–D). Among the first set of YM155 analogs, two YM155 analogs (**5r** and **5s**) with hydroxyl groups induced cell death in hESCs, which was most pronounced with **5s** (Figure S1; Figure 1E). It is noteworthy that YM155 analogs with uncyclized moieties (**4**), unsubstituted benzyl groups (**5c**), and substituted phenyl groups (**5a**, **5b**) showed no activity (Figure S1; Figure 1E).

**Table 1:** Structures of synthesized imidazolium compounds.

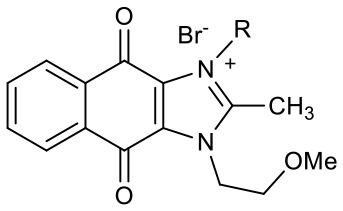								
**Entry**	***R***	**Activity**	**Entry**	***R***	**Activity**	**Entry**	***R***	**Activity**
**5a**	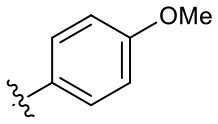	–	**5j**	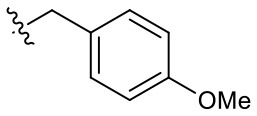	+	**5s**	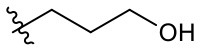	+++
**5b**	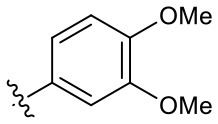	–	**5k**	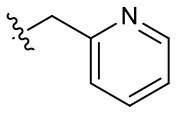	+	**5t**	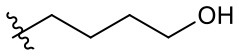	+
**5c**	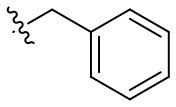	–	**5l**	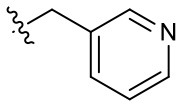	+++	**5u**	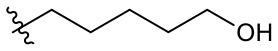	+
**5d**	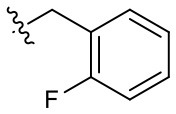	+	**5m**	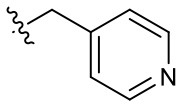	+++	**5v**	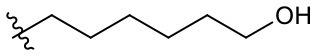	+
**5e**	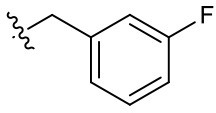	+	**5n**	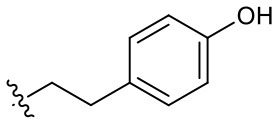	+	**5w**	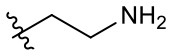	–
**5f**	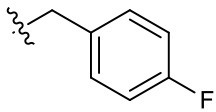	++	**5o**	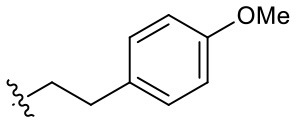	–	**5x**	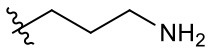	–
**5g**	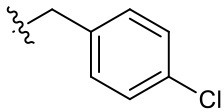	–	**5p**	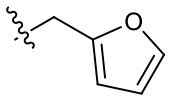	+	**5y**	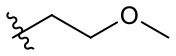	+
**5h**	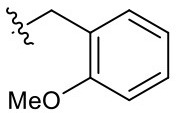	–	**5q**	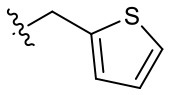	–	**5z**	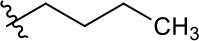	–
**5i**	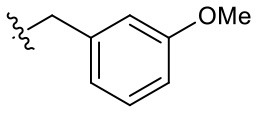	–	**5r**	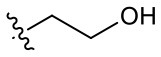	++			

### Hydrogen Bond Acceptor Distance Markedly Affects Stemotoxic Activity

**Figure 2:**
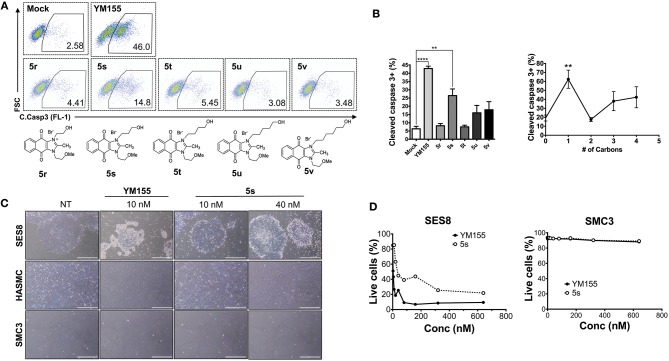
Hydrogen bond acceptor distance markedly affects stemotoxic activity **(A)** FACS analysis for cleaved caspase3 after treatment of YM155 and YM155 analogs (50 nM), Chemical structures of YM155 analogs were presented below. **(B)** Percentage of cleaved caspase3-positive cells at indicative YM155 analogs (left) and number of carbon chain of alcohol group of each compound (right) (*n* = 4) was presented as bar graph (*P* < 0.01 (**), 0.0001 (****)). **(C)** Light microscopic images of hiPSC and differentiated cells (HASMC and SMC3) 24 h after treatment of indicated compounds (Scale bar = 300 μm). **(D)** Percentages of live cells (Annexin-V ^negative^ and 7-AAD ^negative^ population) after treatment of YM155 and **5s** in hiPSCs (SES8: left) and SMC3 (right) were presented.

Given that **5s**, possessing a 3-hydroxypropyl group, appeared more potent than **5r**, possessing a 2-hydroxyethyl group (Figure 1E), we altered the carbon chain harboring the hydroxyl group and examined the stemotoxic activity. Using this approach, we synthesized three more YM155 analogs with carbon chains of differing length (from two to six). As shown in Figure 2B, stemotoxic activity was dramatically attenuated in YM155 analogs with more than three carbons in the chain, suggesting that the presence of the hydroxyl group at a certain distance from the imidazolium ring structure may be important for stemotoxic activity. Interestingly, YM155 analogs, with an amine group (**5w** and **5x)** instead of a hydroxyl group at the same distance (Figure S2), displayed only weak or no stemotoxic activity. Given that the nitrogen atom in amine groups is a much weaker hydrogen bond acceptor than the oxygen atom, this result is consistent with our hypothesis. Additionally, YM155 analogs with ammonium salts present as protonated forms at physiological pH and can in principle serve as hydrogen bond donors rather than hydrogen bond acceptors, owing to the high pKa value (~10–11) of the conjugate acids of aliphatic amine groups when present at the same distance as hydroxyl groups. Accordingly, we hypothesized that the presence of a hydrogen bond acceptor at a specific distance may be crucial for stemotoxic activity. Next, we examined whether **5s** retained stemotoxic activity by screening against isogenic human aortic smooth muscle cells (HASMCs), iPSCs from HASMCs (SES8 cells), and SMCs derived from SES8 cells (SMC3 cells), as described previously (Lee et al., 2013). Similar to YM155, **5s** induced selective cell death of human iPSCs (SES8 cells) in a dose-dependent manner, but not differentiated smooth muscle cells (HASMCs or SMC3s; Figure 2D), although stemotoxic activity was weaker than that of YM155.

### Nitrogen in the Imidazolium Ring of YM155 Is a Hydrogen Bond Acceptor, and Crucial for Stemotoxic Activity

**Figure 3:**
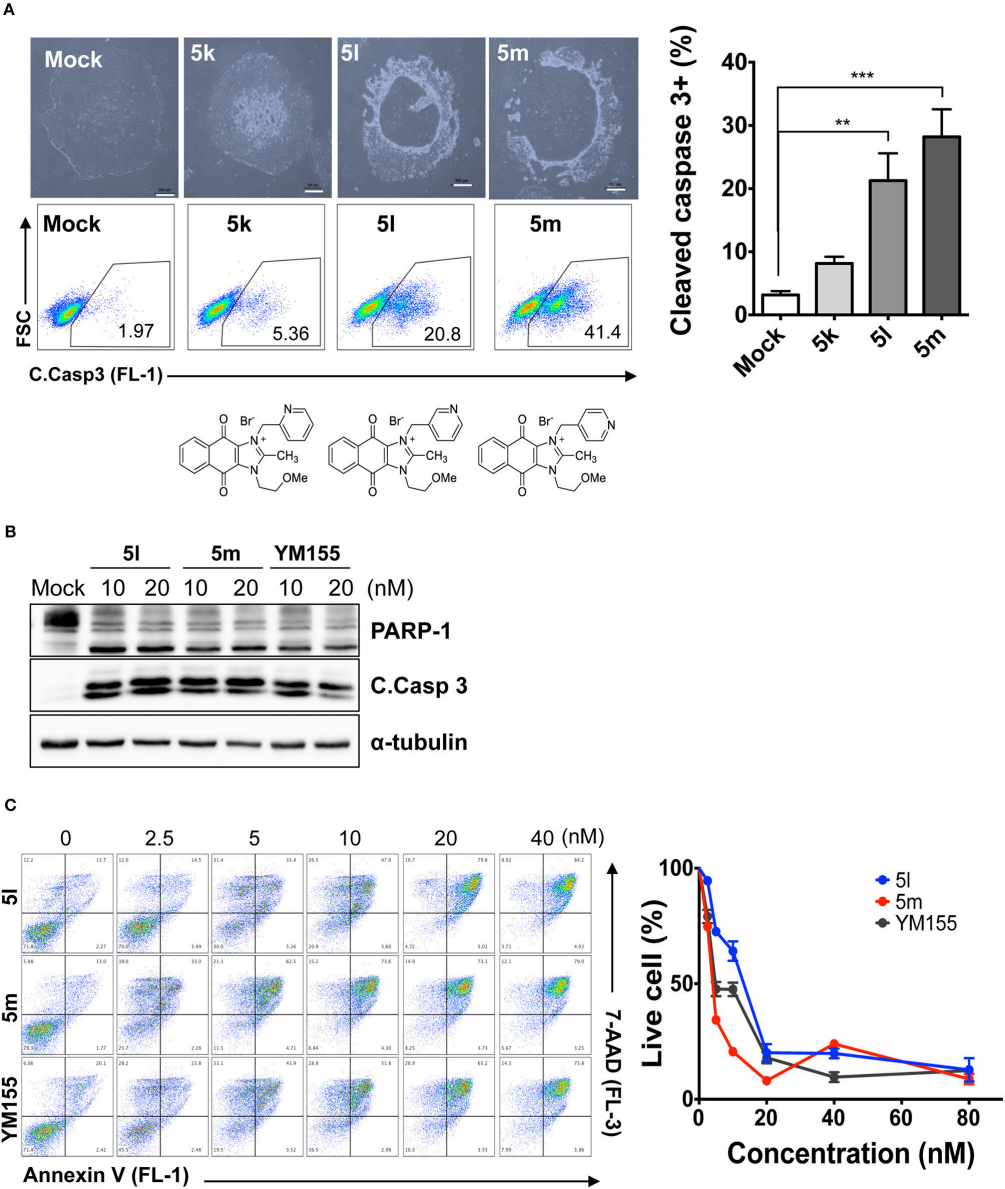
Nitrogen in the pyrazine ring of YM155 is a hydrogen bond acceptor, and crucial for stemotoxic activity **(A)** Light microscopic images (top) and FACS analysis for cleaved caspase3 staining after treatment of indicated compounds (**5k**, **5l**, and **5m**, 50 nM) (bottom) with chemical structure, Percentages of cleaved caspase3 positive cells were presented as bar graph (right) (*n* = 4) (Scale bar = 300 μm) (*P* < 0.01 (**), 0.001 (***)). **(B)** Immunoblotting analysis for PARP-1, cleaved caspase 3 (C.Casp 3) after treatment of indicative dose of compounds, α-tubulin for equal protein loading control. **(C)** FACS analysis for Annexin-V/7-AAD after indicative dose of YM155 and YM155 analogs in hESCs (left), Percentages of live cells were graphically presented (right).

Since the hydroxyl group in **5s** is located three carbons away from the imidazolium moiety, and is a putative hydrogen bond acceptor important for stemotoxic activity, we speculated that one of the two nitrogens in the pyrazine ring of YM155 may serve as a hydrogen bond acceptor and may also contribute to stemotoxic activity. To test this, **5c** lacking the two nitrogens in the aromatic ring was prepared, and as expected, stemotoxic activity was completely lost (Figure 1E). Thus, we next synthesized three more YM155 analogs with nitrogen located at different positions (ortho, meta, and para) of the aromatic ring, and tested the effect on hESCs. Strikingly, **5l** with one nitrogen atom in the meta position exhibited greater stemotoxic activity than **5s**, with a 3-hydroxylpropyl group serving as a hydrogen bond acceptor (Figure S3A). As shown in Figure 3A, **5m** with a nitrogen in the para position of the aromatic ring was more effective toward hESCs (Figure 3A) than a nitrogen in the ortho (**5k**) or meta (**5l**) positions (Figure S3B). We noticed that both **5l** (meta) and **5m** (para) induced cell death of hESCs comparably to YM155 when the dose was >10 nM (Figure 3B). However, **5m** appeared to be more potent than **5l** below the 5 nM range, and equivalent to YM155 (Figure 3C).

### Stemotoxic Activity of 5m

**Figure 4:**
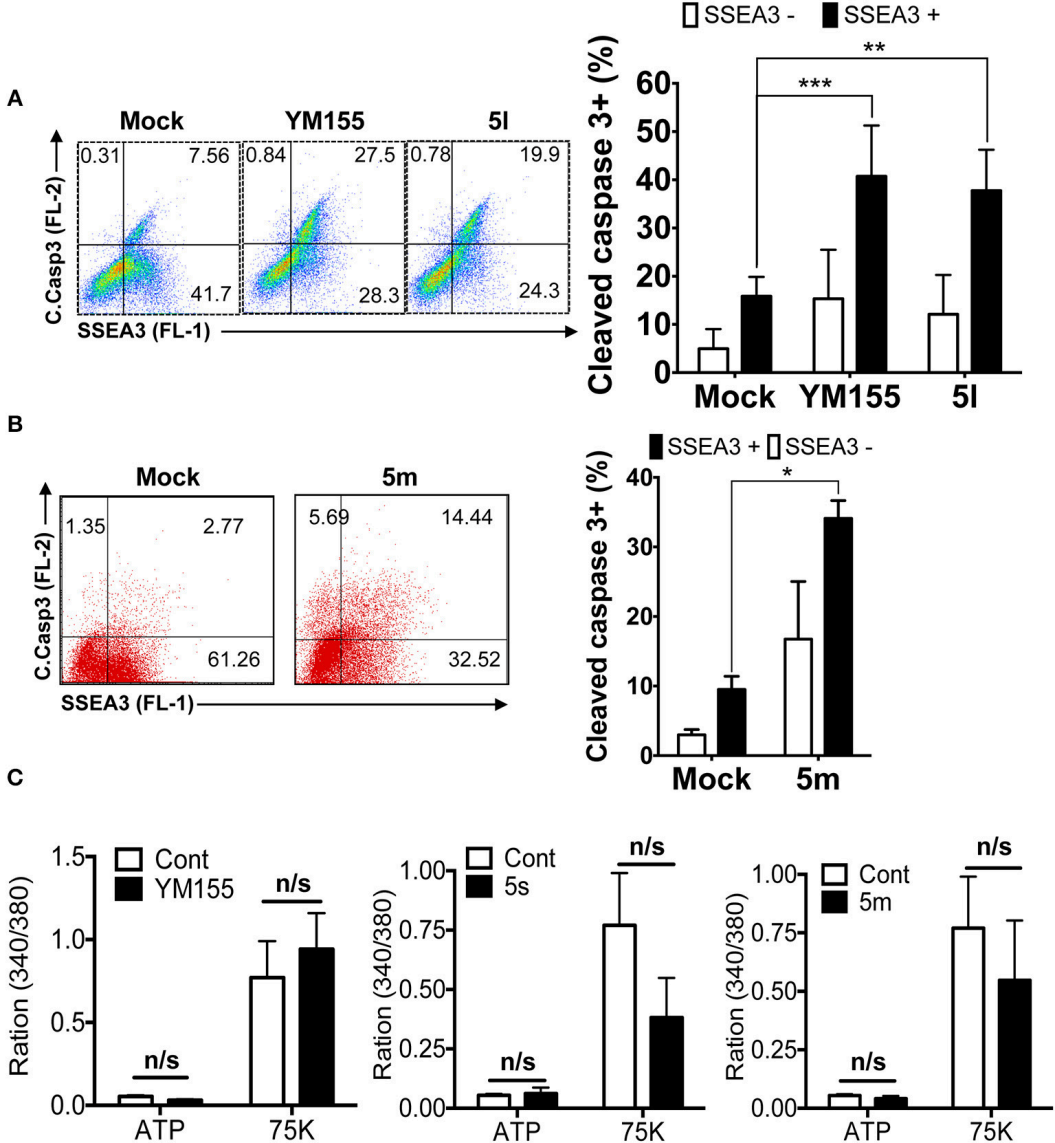
Stemotoxic activity of **5m (A)** FACS analysis for cleaved caspase3 and SSEA3 staining after treatment of YM155 or **5l** (left), Percentages of cleaved caspase3^+^ cells were presented by bar graph (right) (*n* = 4) (*P* < 0.01 (**), 0.001 (****)). **(B)** FACS analysis for cleaved caspase3 and SSEA3 staining after treatment of **5m**, Percentages of cleaved caspase3^+^ cells were presented by bar graph (right) (*n* = 4) (*P* < 0.05 (*)). **(C)** Net changes in intracellular calcium levels in response to a pharmacological agonist, ATP (20 μM) or membrane depolarization (75 mM K+) after treatment of indicated chemicals in the SMC3 cells.

**Figure 5:**
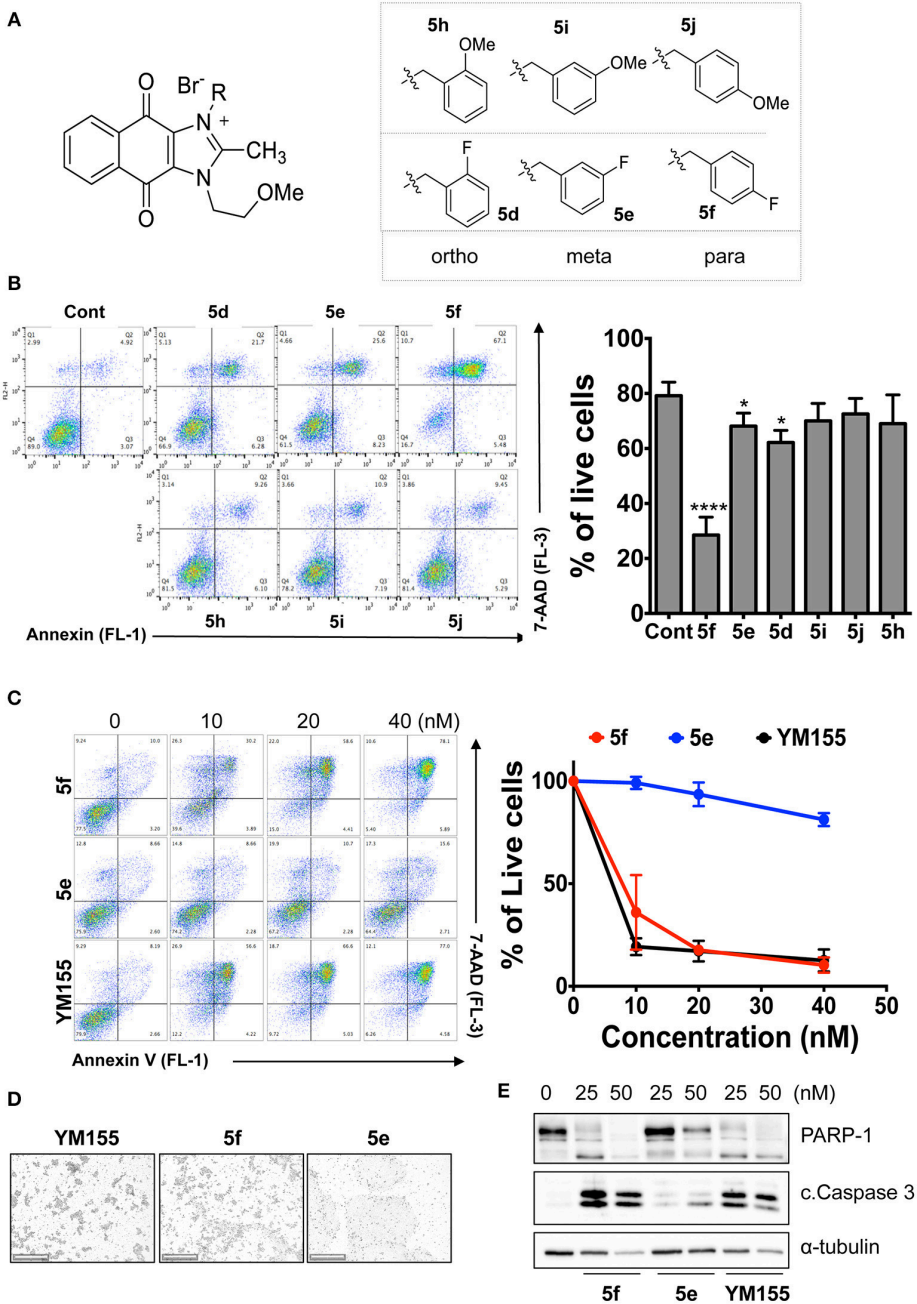
Hydrogen bond acceptors strongly influence the stemotoxic activity of YM155 analogs **(A)** Chemical backbone structure of YM155 analogs (left) and six YM155 analogs (right). **(B)** FACS analysis for Annexin-V/PI staining after treatment of indicated compounds, Percentages of live cells (Annexin-V ^negative^ and PI ^negative^ Cells) were presented as bar graph (right) (*n* = 6) (*P* < 0.05 (*), 0.0001 (****)). **(C)** FACS analysis for Annexin-V/7-AAD staining after indicative concentration of YM155 and YM155 analogs (**5e** or **5f**) (left). Percentages of live cells (Annexin-V ^negative^ and 7-AAD ^negative^ Cells) were presented as bar graph (right). **(D)** Light microscopic images after treatment of 20 nM of YM155 and YM155 analogs (Scale bar = 500 μm). **(E)** Immunoblotting analysis for PARP-1 and cleaved caspase 3 (C.Casp3) after indicative concentration of YM155 analogs, α-tubulin for equal protein loading control.

To safely apply stemotoxic agents that ablate undifferentiated hPSCs, differentiated cells derived from hPSCs should remain unaffected after treatment, while undifferentiated hPSCs should undergo selective cell death (Jeong et al., 2017a). To test this, we examined the reactivity of YM155 analogs showing moderate to high stemotoxic activity (e.g., **5l** and **5m**) toward differentiated cells derived from hPSCs. As shown in Figures 4A,B, both **5l** (Figure 4A) and **5m** (Figure 4B) induced cell death only in SSEA3-positive populations (i.e., undifferentiated hPSCs) but not SSEA3-negative populations (differentiated cells derived from hPSCs) when used to treat partially differentiated hPSCs (a mixture of differentiated and undifferentiated cells). Furthermore, the functionality of SMCs derived from iPSCs (Lee et al., 2010, 2013), determined by Ca^2+^ influx (Lee et al., 2010), remained unaltered after treatment with **5s** or **5m**, similar to YM155 (Figure 4C). These results clearly suggest that **5m** may be applicable as a stemotoxic compound.

### Hydrogen Bond Acceptors Strongly Influence the Stemotoxic Activity of YM155 Analogs

Given that **5m** with a nitrogen at the para position showed stemotoxic activity equivalent to YM155, we speculated that a hydrogen bond acceptor at that position may be important for interaction with the target protein, and hence may be critical for stemotoxic activity. To test this, we synthesized six YM155 analogs with hydrogen bond acceptors at different positions on the benzene ring (Figure 5A), and screened them against hPSCs. As shown in Figures 5B,C, **5f**, which mimics **5m** and has a hydrogen bond acceptor at the para position, displayed clear stemotoxicity, while **5d** (mimicking **5k**) or **5e** (mimicking **5l**) exhibited only moderate stemotoxic activity (Figures 5B,C). It is noteworthy that each analog with a methoxy group **(5h**, **i**, and **j)** lost all activity (Figure 5B). The unexpectedly low stemotoxic activity of **5h**–**j**, even when a hydrogen bond acceptor (methoxy substituent) was present, is likely due to steric hindrance of the adjacent methyl group, which might interfere with interactions between small molecules and target proteins. Additionally, the results of fluoride substitution, which can also function as a hydrogen bond acceptor rather than a hydrogen bond donor, support this proposal. Compound **5f** showed higher stemotoxic activity than **5e**, as determined by cellular morphology (Figure 5C) and immunoblotting of apoptotic markers (PARP-1 cleavage and formation of active caspase 3; Figure 5E), suggesting that the location of the hydrogen bond acceptor is critical for stemotoxic activity via recognition by the target protein.

### SLC35F2 Is Required for Cell Entry of YM155 Analogs and Hence Stemotoxic Activity

**Figure 6:**
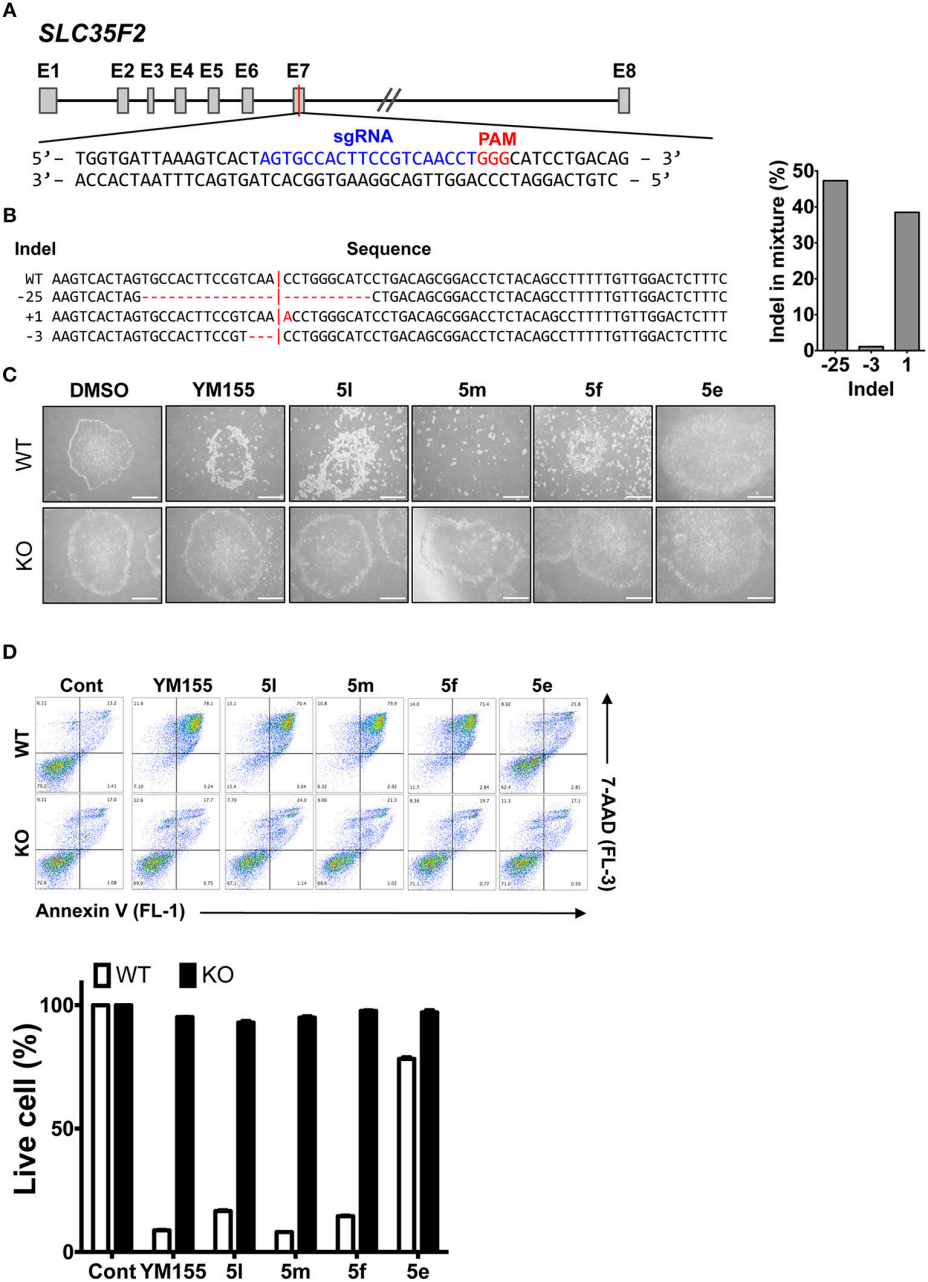
SLC35F2 is required for cell entry of YM155 analogs and stemotoxic activity **(A)** Scheme for knock out (KO) of SLC35F2 targeting Exon 7. **(B)** Percentage of mutations quantified from Sanger sequencing and relative contribution of each sequence of wild type (WT) and SLC35F2 KO hESCs was graphically presented. **(C)** Light microscope images of hESCs (WT) and SCL35F2 knock out hESCs (KO) after treatment of 20 nM of YM155 and YM155 analogs (Scale bar = 300 μm). **(D)** FACS analysis for Annexin-V/7-AAD staining after indicative concentration of YM155 in WT or KO (left), Percentages of live cells (Annexin-V ^negative^ and 7-AAD ^negative^ Cells) were presented as a bar graph (right) (*n* = 3).

Based on the role of the solute carrier protein SLC35F2 in the import of YM155 (Winter et al., 2014), and differences in the cytotoxicity of YM155 toward different cancer cell types (Winter et al., 2014), we hypothesized that the stemotoxic activity of YM155 and its analogs may be mediated by SLC35F2. To explore this, cell death of hESCs after treatment with various stemotoxic YM155 analogs was compared with control hESCs (wild type: WT) and SLC35F2 knockout (KO) hESCs generated using the CRISPR/CAS9 system (Figures 6A,B and Figure S4; Kim et al., 2019). As predicted, the stemotoxic activity of YM155 and its analogs was completely lost in KO hESCs, while cell death was evident in WT hESCs (Figures 6C,D). Considering the pivotal role of SLC35F2 for import of YM155 into cells (Winter et al., 2014), the chemical structure of YM155 analogs (e.g., the position of the hydrogen bond acceptor in the aromatic ring) may be important for interaction with SLC35F2 in hESCs.

### Structure-Activity Relationships of YM155 Analogs

**Figure 7:**
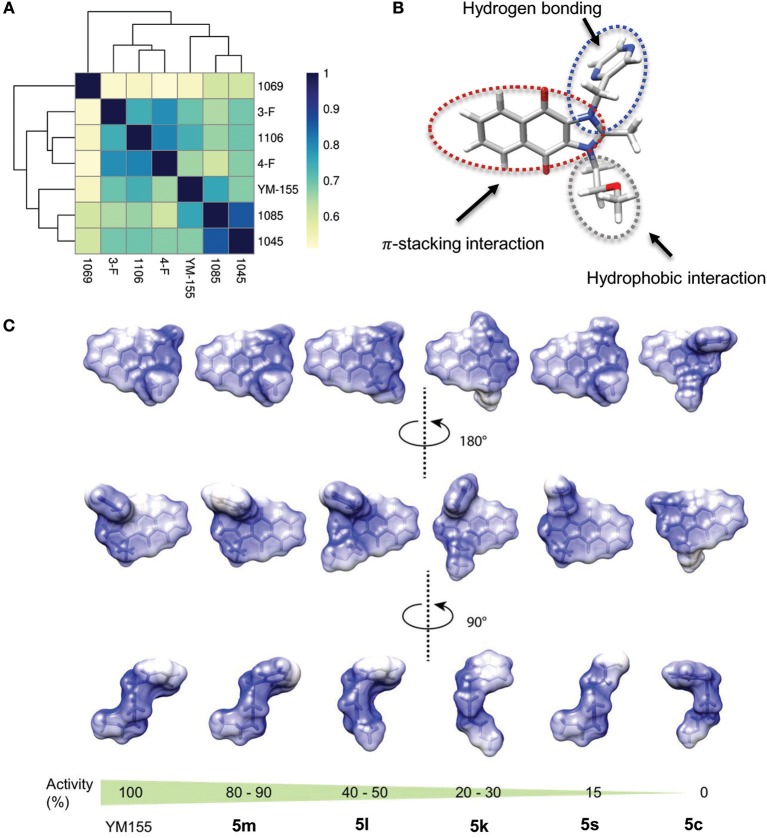
Structure-activity relationships of YM155 analogs. **(A)** Correlation analysis of Tanimoto distance of YM155 analogs. **(B)** Energy minimized 3D structure of YM155 with stick representation method colored by gray (backbone), white (proton), nitrogen (blue), and red (oxygen), respectively. **(C)** Electrostatic surface potential of YM155 analogs was colored by blue (positively charged region) and red (negative charged region) with ±5 scaled energy kcal/(mole*e) unit.

Using Tanimoto (or Jaccard) analysis of molecular similarity (Maggiora et al., 2014), the structural similarity between YM155 analogs displaying stemotoxic activity and YM155 was compared. Compound **5f** has a similar structure to that of **5m** (Figure 7A). Given that the stemotoxic activity of **5m** and **5f** was equivalent to that of YM155 (Figures 3, 5), whereas the stemotoxic activity of **5l** (or **5k**) and **5e** (or **5d**) was not, the presence of a hydrogen acceptor (e.g., nitrogen or fluoride) at the para position, and not the ortho or meta position, may be favorable for the formation of hydrogen bonds with the SLC35F2 target protein (Figure 7B). The structure of YM155 can be divided into three parts in 3D space; a dioxonaphtho-imidazolium part occupying the largest space, a pyrazine aromatic ring connected to the imidazolium ion, and a linear methoxyethyl chain also connected to imidazolium ion. A surface model shows that the pyrazine ring and methoxyethyl chains are attached to opposite sides of the flat dioxonaphtho-imidazolium backbone in YM155 (Figure 7B). Comparison of the surface models and activities of YM155 analogs showed that compounds with a shape that differed from that of YM155 were less active than YM155. For instance, the activity of analogs, in which the pyrazine ring and methoxyethyl chain point in the same direction as the side chain of the dioxonaphtho-imidazolium backbone (**5l**, **5k**, and **5d**), displayed < 30% of the activity of YM155 (Figure 7C). In addition, analogs adopting the same spatial conformation as YM155 but without aromatic rings exhibited low activity (**5s**). Electrostatic surface potential analysis revealed that the 3D structure and surface charge distribution of **5m** are the most similar to YM155 among all analogs (Figure 7C). SAR analysis using surface models suggested that YM155 analogs should maintain the aromatic ring and the hydrophobic chain in opposite orientations around the dioxonaphtho-imidazolium backbone to possess sufficient stemotoxic activity. Although the precise active site or pharmacophore to which YM155 analogs can bind remains unknown, the specific 3D (conformational) properties of YM155 analogs are likely to make a crucial contribution to interactions with the target protein, along with π-π stacking, hydrogen bonding, and hydrophobic interactions.

## Discussion

As clinical trials of hPSC-based cell therapies are extended worldwide (Kimbrel and Lanza, 2015), associated risk factors, especially tumorigenicity, must be completely resolved to ensure safety (Heslop et al., 2015). Among various approaches for selective elimination of tumorigenic hPSCs (Jeong et al., 2017a), efficacy of teratoma inhibition, by relatively low dose of YM155 (at nM range), was determined by multiple independent studies (Bedel et al., 2017; Kang et al., 2017; Kim K. T. et al., 2017). Thus, SAR analysis of YM155 is important for developing more potent stemotoxic analogs. To this end, in the present work we synthesized 26 YM155 analogs with modified pyrazine ring structures, and found that the positions of hydrogen bond acceptors in the aromatic ring and the imidazolium ring are crucial for interactions with the target protein (possibly SLC35F2). The distance between the hydrogen bond acceptor and the imidazolium ring is one factor that is determinant of stemotoxic activity (Figure 2), and a nitrogen located in the meta or para position appears to be desirable (Figure 3, Figure S3). Similar results were obtained with **5e** and **5f**, in which substitution of a fluoride with a sterically unfavorable methoxy group (**5h−5j**) resulted in the complete loss of activity (Figure 5) due to the high sensitivity to steric interactions. SLC35F2, a putative target protein of YM155, was shown to be responsible for uptake of YM155 in a cancer cell model (Winter et al., 2014). Because knockout of SLC35F2 in hESCs completely prevented hESC cell death by YM155 and its analogs (Figure 6), we speculate that hydrogen bonding interactions between YM155 and SLC35F2 may be critical for YM155 uptake, and consequentially for cytotoxic activity against both cancer cells and hPSCs. Since the 3D structure of SLC35F2 remains undetermined, precise determination of exactly how hydrogen bond acceptors on the aromatic ring structure may contribute to protein-ligand interactions cannot be achieved at present. For future application of YM155 and its analogs as both stemotoxic compounds and anti-cancer drugs (Nakahara et al., 2007; Clemens et al., 2015), it is important to determine the structure of SLC35F2 to facilitate the design of more potent YM155 analogs with increased uptake, and hence enhanced stemotoxic activity.

## Conclusion

In summary, we synthesized total 26 YM155 analogs and demonstrated that nitrogen in the pyrazine ring can serve as a critical hydrogen bond acceptor, and these interactions are crucial for stemotoxic activity. Through structure-activity relationship (SAR) analysis, we developed a novel YM155 analog with a flurobenzyl group that has stemotoxic activity equivalent to that of YM-155. Additionally, we showed that the formation of hydrogen bonds involving the pyrazine ring structure is important for recognition of YM155 by SLC35F2, a membrane solute carrier protein, to induce cell death. Furthermore, analysis of the electrostatic surface potential supported the SAR evaluation of YM155 analogs based on 3D conformations.

## Author Contributions

CL and SC performed the chemical synthesis under supervision of Y-GS and S-HK. NMR, IR, LRMS, and HRMS were performed by CL and SC under supervision of Y-GS and S-HK. hPSCs and differentiated cell culture, Cell death assay, and Immunoblotting assay were performed by Y-HG and H-CJ under the supervision of M-OL and H-JC. HL and WK performed Tanimoto analysis. Energy minimized 3D structure and electrostatic surface potential of YM155 analogs were generated by O-SK and WS. The manuscript was written by H-JC and S-HK with the support of WS, Y-GS, WK, and M-OL. All authors approved the manuscript in its final form for publication.

### Conflict of Interest Statement

The authors declare that the research was conducted in the absence of any commercial or financial relationships that could be construed as a potential conflict of interest.
