# Supplementary material for: Structure-Activity Relationship Analysis of YM155 for Inducing Selective Cell Death of Human Pluripotent Stem Cells
Source: Front Chem. 2019 May 16;7:298. doi: 10.3389/fchem.2019.00298 (PMC6532689; doi:10.3389/fchem.2019.00298)
Supplement: Supplementary file 1 [file Data_Sheet_1.docx]

Supplementary Material

**Figure S1. Screening of stem-toxic activity of YM155 analogs** Light microscope images of hiPSC (SES8) after treatment of YM155 (10 or 50 nM) and 50 nM of YM155 analogs

**Figure S2.** **Stem-toxic activity of YM155 analogs with aliphatic amino group** (A) Light microscope images of hiPSC (SES8) after treatment of YM155 and YM155 analogs (50 nM), Chemical structure of **5w** and **5x** presented below (B) FACS analysis for Annexin-V / 7-AAD staining

**Figure S3. Stem-toxic activity of hydrogen bond acceptor in imidazolium ring of YM155** (A) Light microscope images of hiPSC (SES8) after treatment of YM155, **6**, **5s**, **5l** (40 nM or 80 nM). (B) Chemical structure of YM155 and three YM155 analogs (**5k**, **5l** and **5m**) with different position of hydrogen acceptor (nitrogen) used in this study. Stem-toxic activity of each YM155 analog was presented as number of + (e.g., +++: high, ++: moderate, +: weak, -: none).

**Figure. S4**

**PAM**

**Figure S4. Knock out of SLC35F2 in hESCs** Sanger sequencing of wild-type (WT) and SLC35F2 knock out (KO) in H9.
